# Patient–ventilator asynchrony during conventional mechanical ventilation in children

**DOI:** 10.1186/s13613-017-0344-8

**Published:** 2017-12-20

**Authors:** Guillaume Mortamet, Alexandrine Larouche, Laurence Ducharme-Crevier, Olivier Fléchelles, Gabrielle Constantin, Sandrine Essouri, Amélie-Ann Pellerin-Leblanc, Jennifer Beck, Christer Sinderby, Philippe Jouvet, Guillaume Emeriaud

**Affiliations:** 1 Pediatric Intensive Care Unit, CHU Sainte-Justine, 3175 Côte Sainte-Catherine, Montreal, QC Canada; 2 INSERM U 955, Equipe 13, Créteil, France; 3 CHU Sainte-Justine Research Center, Université de Montréal, Montreal, Canada; 4 Pediatric Intensive Care Unit, CHU Fort-de-France, Fort-de-France, France; 5 Department of Pediatrics, CHU Sainte-Justine, Montreal, QC Canada; 6 Queen’s University, Kingston, Canada; 7 Keenan Research Centre for Biomedical Science, Li Ka Shing Knowledge Institute, St. Michael’s Hospital, Toronto, ON Canada; 8 Department of Pediatrics, University of Toronto, Toronto, Canada; 9 Institute for Biomedical Engineering and Science Technology (iBEST), Ryerson University and St-Michael’s Hospital, Toronto, Canada; 10 Department of Medicine, University of Toronto, Toronto, Canada

**Keywords:** Diaphragm function, Mechanical ventilation, Patient–ventilator asynchrony, Patient–ventilator interaction, Pediatric intensive care unit, Pediatrics

## Abstract

**Background:**

We aimed (1) to describe the characteristics of patient–ventilator asynchrony in a population of critically ill children, (2) to describe the risk factors associated with patient–ventilator asynchrony, and (3) to evaluate the association between patient–ventilator asynchrony and ventilator-free days at day 28.

**Methods:**

In this single-center prospective study, consecutive children admitted to the PICU and mechanically ventilated for at least 24 h were included. Patient–ventilator asynchrony was analyzed by comparing the ventilator pressure curve and the electrical activity of the diaphragm (Edi) signal with (1) a manual analysis and (2) using a standardized fully automated method.

**Results:**

Fifty-two patients (median age 6 months) were included in the analysis. Eighteen patients had a very low ventilatory drive (i.e., peak Edi < 2 µV on average), which prevented the calculation of patient–ventilator asynchrony. Children spent 27% (interquartile 22–39%) of the time in conflict with the ventilator. Cycling-off errors and trigger delays contributed to most of this asynchronous time. The automatic algorithm provided a NeuroSync index of 45%, confirming the high prevalence of asynchrony. No association between the severity of asynchrony and ventilator-free days at day 28 or any other clinical secondary outcomes was observed, but the proportion of children with good synchrony was very low.

**Conclusion:**

Patient–ventilator interaction is poor in children supported by conventional ventilation, with a high frequency of depressed ventilatory drive and a large proportion of time spent in asynchrony. The clinical benefit of strategies to improve patient–ventilator interactions should be evaluated in pediatric critical care.

## Background

Mechanical ventilation is commonly used in pediatric intensive care units (PICUs) [1]. Maintaining the patient’s own spontaneous breathing effort during ventilation is key. Assisted (or patient-triggered) ventilation may improve ventilation perfusion matching and forestall the development of ventilator-induced diaphragmatic dysfunction [2]. As the patient contributes in the ventilation, good interaction between the patient and the ventilator is essential.

Children have higher respiratory rates, smaller tidal volumes, and weaker inspiratory efforts when compared with adults, and patient–ventilator synchrony is difficult to achieve in pediatric patients [3]. These can lead to a mismatch between the patient and the ventilator, defined as a patient–ventilator asynchrony (PVA). PVA includes the inspiratory and expiratory timing errors (delays between patient demand and ventilator response), efforts undetected by the ventilator, assist delivered in the absence of patient demand, and double triggering (two rapidly successive assists following a single effort).

In critically ill adults, asynchronies occur frequently and are associated with prolonged ventilator support, sleep disorders, poor lung aeration, longer stay in the intensive care unit and mortality [4–9]. Pediatric data in this field are lacking. PVA seems frequent in PICU [10–13], but little is known about the risk factors of PVA and the association with patient outcome.

In the present study, we aimed to describe the characteristics of PVA in critically ill children, to identify risk factors associated with PVA, and to evaluate the association between PVA and patient outcome.

## Methods

This prospective observational study was conducted in the PICU of CHU Sainte-Justine, a university-affiliated pediatric hospital, from August 2010 to October 2012. The study protocol was approved by the ethics committee of CHU Sainte-Justine. Written informed consent was obtained from the parents or legal tutor.

### Patients

Consecutive children aged between 7 days and 18 years admitted to the PICU and mechanically ventilated for at least 24 h were eligible. The screening was performed daily by a research assistant. Eligible patients reached inclusion criteria when the presence of spontaneous breathing was evidenced by clinical respiratory efforts or by a respiratory rate sustainably higher than the set ventilator rate. Patients were excluded if they had one of the following criteria: chronic respiratory insufficiency with prior ventilatory support longer than 1 month, tracheostomy, neuromuscular disease, contraindications to nasogastric tube exchange (i.e., local trauma, recent local surgery, or severe coagulation disorder), suspected bilateral diaphragm paralysis, immediate postcardiac surgery period, expected death in the next 24 h, or a limitation of life support treatment.

No modification of the ventilator settings was done for the study. The attending physicians set the ventilator mode and settings according to the local practices. Patients were ventilated with the Evita XL (Dräger, Lubeck, Germany) or the Servo-I ventilator (Maquet, Solna, Sweden). Sedation and analgesia were decided by the treating team and usually involved a combination of benzodiazepines and opioids. There was no local written protocol regarding the ventilator management or the sedation during the study. The ventilation support was reassessed every 1 or 2 h by respiratory therapists according to local practice. At the time of the study, neurally adjusted ventilatory assist (NAVA) was not routinely used in clinical practice in our unit.

### Protocol

PVA was recorded at two different times during the PICU stay. We obtained a first 30-min recording in acute phase, i.e., as soon as possible after inclusion in the study, and an esophageal catheter was installed to record the electrical activity of diaphragm (Edi). The second (pre-extubation) recording was performed during 15 min in the 4 h preceding extubation, if the Edi catheter was still in place.

### Data recording

PVA was analyzed by comparing the ventilator pressure curve and the Edi signal. Edi was recorded using a specific nasogastric catheter (Edi catheter, Maquet, Solna, Sweden) connected to a dedicated Servo-I ventilator (Maquet, Solna, Sweden). This ventilator was used only to continuously process and record the Edi signal, the patient being ventilated with his own ventilator as before the study. The catheter was positioned according to the recommendations of the manufacturer as previously described [12, 14].

Demographic data and patient’s characteristics, including age, gender, weight, time of measurements, admission diagnostic and comorbidities, Pediatric Index of Mortality (PIM) II and Pediatric Logistic Organ Dysfunction (PELOD) scores, were collected. The sedation score was calculated for the 4-h period preceding the first recording, as suggested by Randolph et al. [15], using a score for which one point was given for the amount of each drug that would be equivalent to 1 h of sedation in a nontolerant subject. The Comfort B scale was used to determine the level of comfort (comfort is better when score is lower).

### Clinical outcomes

The primary outcome was the number of ventilator-free days at day 28 (since intubation). Patients who died were considered having zero ventilation-free day. The secondary clinical outcomes were first extubation success (no need for invasive ventilation support within 48 h of extubation), duration of mechanical ventilation, and length of PICU stay.

### PVA manual analysis

As previously described [12, 16, 17], for each recording, Edi and ventilator pressure curves were analyzed in a breath-by-breath manner over a continuous 5-min period exempt of artifacts linked to agitation or patient care. Timings of the beginning and the end of inspiration and expiration phases on the Edi and the ventilatory pressure signals were semiautomatically identified: Main timings were automatically identified, and a visual inspection was performed breath by breath, permitting to validate and/or adjust the timing cursors if necessary. All analyses were performed by two independent investigators. By comparing the ventilator and Edi timings, PVA was identified, including wasted efforts (clear effort observed on Edi with no ventilator assist), auto-triggered breath (ventilator assist delivered in the absence of Edi increase), double triggering (two rapidly successive assists following a single effort), and inspiratory trigger and cycling-off errors. As the response of the ventilator for triggering or cycling off could be frequently either retarded or premature [12], we reported both types of asynchrony.

The main PVA variable of interest was the percentage of time spent in asynchrony, calculated from the total duration spent in each type of PVA (wasted efforts, auto-triggering, double triggering, trigger and cycling off errors) divided by the duration of the recording. A priori, we defined severe PVA when the percentage of time spent in asynchrony was superior to the 75th percentile of the entire cohort, i.e., the quarter of patients with the worst synchrony.

In order to facilitate the comparison with other studies [18], we also calculated the asynchrony index (AI), defined as the number of asynchronous events (i.e., the sum of wasted efforts, ineffective triggering, double triggering, and cycles with important trigger and cycling-off errors) divided by the total respiratory rate (i.e., the sum of ventilator cycles and wasted efforts), and expressed as a percentage. Important trigger and cycling-off errors were considered when the error (i.e., premature or delayed response) exceeded 33% of inspiratory and expiratory times, respectively. An AI > 10% was considered as a high incidence of asynchrony [5, 18].

### PVA automatic analysis

Asynchrony was also analyzed using a standardized automated method over the same period, to prevent interobserver variability and to avoid observer subjectivity [19]. Inspiratory and expiratory timings were fully automatically detected on ventilator pressure and Edi signals based on predetermined thresholds (0.5 μV for Edi amplitude). Asynchrony was quantified using the NeuroSync index, a global index considering both inspiratory and cycling-off errors. A higher NeuroSync index reflects worse asynchrony, and synchrony can be considered as poor when NeuroSync index exceeds 20% [20, 21].

### Sample size calculation

Based on studies conducted in adults, we expected a difference in ventilator-free days of 6 days. With a group distribution of 3/1 and a type-1 error risk of 0.05, the inclusion of 56 patients was necessary to achieve a power of 80%. We planned to enroll a sample of 60 patients to take into account the attrition risk.

### Statistical analysis

Data are expressed as median values (with interquartiles, IQR) for continuous variables, and number and/or frequency (%) for categorical data. Differences in categorical variables were tested using Chi-square or Fisher’s exact test. Differences in continuous variables were assessed by the nonparametric Mann–Whitney test, the paired *t* test, or the Wilcoxon test.

Patients with peak inspiratory Edi < 2 µV were a posteriori excluded from PVA analysis (both manual and automated) because the reality of the spontaneous activity in those patients appeared questionable, and the identification of PVA is complex. intraclass correlation coefficient (ICC, two-way random model) was calculated to assess interobserver reproducibility for manual PVA analysis and to compare the results from the manual and the automatic methods. After confirmation of an excellent interobserver agreement (ICC > 0.75), the averages of the two observer’s results were calculated and used in further analysis.

The association of potential risk factors with severe PVA was studied by univariate logistic regression analysis. Noncollinear factors associated with a univariate association with p < 0.05 were included in a multivariate logistic regression. The relationship between PVA and clinical outcomes was described using univariate analysis.

All p values are two-tailed and considered significant if p < 0.05. Statistical analyses were performed using SPSS 24.0 (SPSS, Inc, Chicago, IL).

## Results

### Study population

**Fig. 1:**
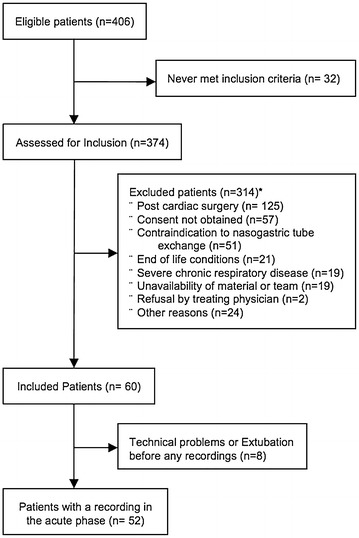
Study flowchart (*patients could be excluded for two reasons)

During the study period, 2090 patients were admitted to the PICU. Among the 406 eligible patients, 60 patients reached inclusion criteria and were enrolled (Fig. 1). Exploitable signals were finally available in 52 patients, who were included in the analysis. Median age of eligible patients who were not included was 8 (1–48) months old, which is similar to analyzed patients (p = 0.96). Twenty-two of these patients also had a second recording in the pre-extubation period. The patient characteristics are presented in Table 1. They were studied 4 (IQR: 1–10) days after PICU admission.

**Table 1:** Characteristics of population (n = 52)

	Total *n* = 52	Peak Edi < 2 µV *n* = 18	Peak Edi > 2 µV *n* = 34
Age (months)	10 (2–42)	21 (1–135)	6 (2–29)
Weight (kg)	6.5 (4.3–17.4)	11 (4.8–38.4)	5.3 (4.0–12.0)
Male, n (%)	31 (60%)	11 (61%)	20 (59%)
Days between admission and inclusion	4 (1–10)	3 (1–7)	4 (1–10)
Days between MV initiation and inclusion	3 (1–7)	2 (1–6)	4 (2–7)
Main reasons for PICU admission, *n* (%)			
Respiratory failure	31 (60%)	5 (28%)	26 (76%)*
Including bronchiolitis	11 (21%)	1 (6%)	10 (29%)
Hemodynamic failure	3 (6%)	2 (11%)	1 (3%)
Neurologic disorder	9 (17%)	6 (33%)	3 (9%)
Metabolic disorder	2 (4%)	0 (0%)	2 (6%)
Trauma	2 (4%)	2 (11%)	0 (0%)
Postoperative admission	5 (10%)	3 (17%)	2 (6%)
Chronic condition, *n* (%)			
Respiratory disease	8 (15%)	2 (11%)	6 (18%)
Cardiac disease	9 (17%)	3 (17%)	6 (18%)
Neurological disease	11 (21%)	4 (22%)	7 (21%)
Immuno-oncologic disease	3 (6%)	0 (0%)	3 (9%)
Clinical status			
PIM-2 score	1.7 (0.8–4.3)	2.3 (0.9–4.5)	1.6 (0.8–4.4)
PELOD score	2 (1–1)	1 (1–11)	1 (1–11)
Set respiratory rate, min^−1^	25 (20–35)	23 (14–38)	31 (25–42)*
Measured respiratory rate, min^−1^	29 (20–36)	20 (15–29)	34 (28–40)*
pH	7.40 (7.35–7.42)	7.40 (7.36–7.43)	7.39 (7.34–7.43)
PaCO_2_, mmHg	46 (42–53)	42 (38–47)	48 (45–57)*
HCO_3_^−^, mmHg	28 (24–32)	27 (23–30)	30 (25–33)
PEEP, cmH_2_O	5 (5–6)	5 (5–5)	5 (5–6)
FiO_2_	0.35 (0.29–0.41)	0.30 (0.24–0.35)	0.35 (0.30–0.50)
Comfort score	13 (10–15)	11 (8–13)	15 (12–16)*
Score sedation	11 (6–21)	10 (1–14)	15 (6–25)
Edi analysis			
Peak inspiratory Edi, µV	3.6 (1.2–7.6)	1.1 (0.6–1.3)	6.6 (3.8–11.5)
Tonic expiratory Edi, µV	0.7 (0.4–1.9)	0.4 (0.3–0.5)	1.1 (0.7–2.5)

Data are expressed as median (interquartile range) or *n* (%)

*Edi* electrical activity of the diaphragm, *MV* mechanical ventilation, *PICU* pediatric intensive care unit, *PEEP* positive end-expiratory pressure

*Significant difference between the two groups (p < 0.05)

Eighteen patients had a very low ventilatory drive (peak Edi < 2 µV on average), which prevented the calculation of PVA. As detailed in Table 1, these patients tended to be older, were affected less frequently by a respiratory disease, and had a lower PaCO_2_ and a lower comfort score as compared to patients with higher drive.

### Magnitude of PVA

**Fig. 2:**
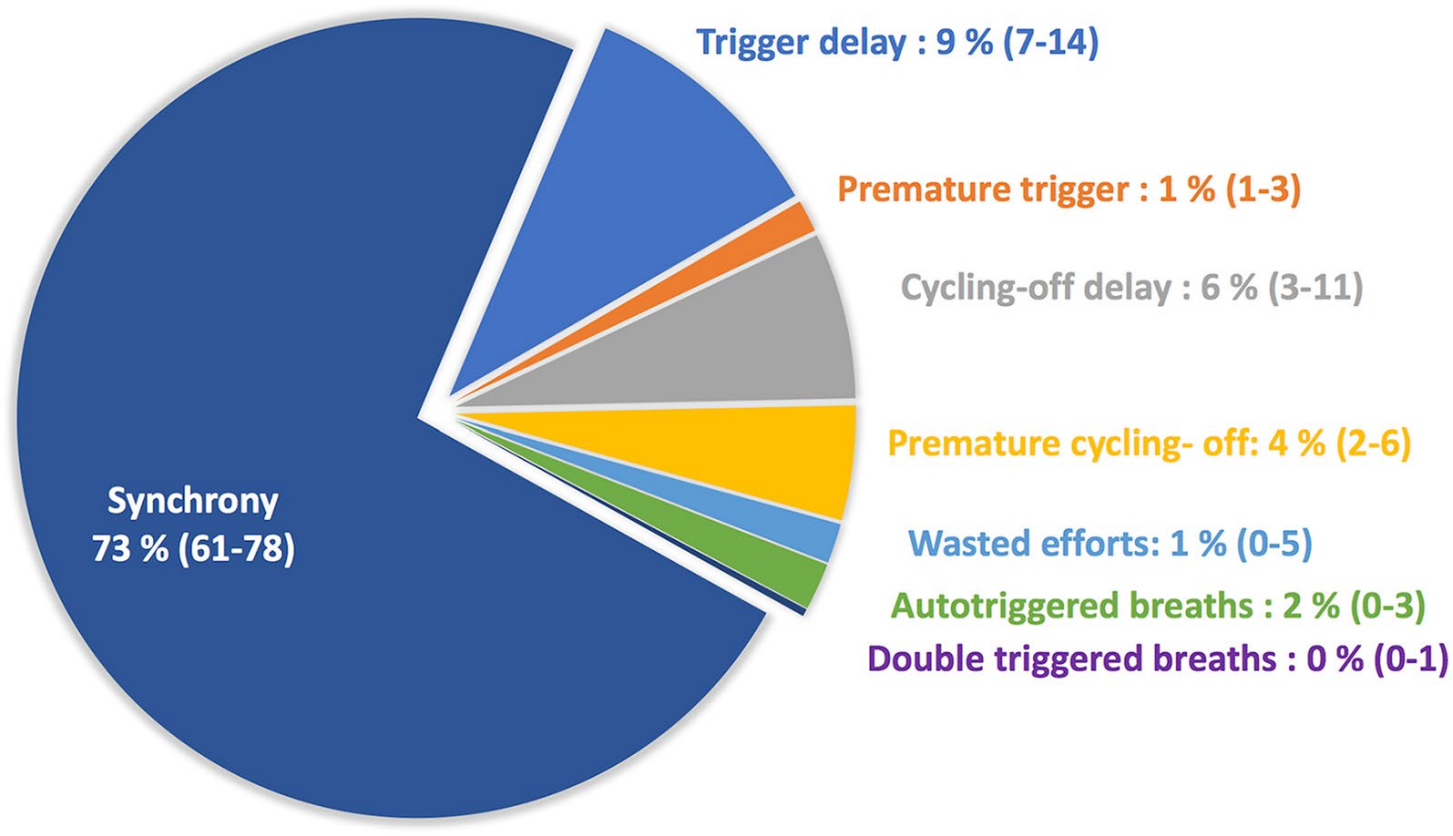
Contribution of the different types of asynchrony in the total time spent in conflict with the ventilator

A total of 9806 breaths were analyzed with the manual method, with a median of 168 (IQR: 123–258) breaths analyzed per recording. The interrater agreement for PVA manual analysis was excellent, with ICC > 0.85 for all PVA parameters. The total proportion of time spent in PVA was 27% (IQR: 22–39) of the time. As illustrated in Fig. 2, cycling-off errors and trigger delays contributed to most of this asynchronous time 12% (IQR: 8–15) and 11% (IQR: 8–16), respectively. Auto-triggered cycles, wasted efforts, and double triggering were also highly prevalent, with two (IQR: 0–3), two (IQR: 1–10), and one (IQR: 0–5) events per minute, respectively.

The median AI was 25% (IQR: 18–35), and 33 (97%) patients had an AI greater than 10%.

### Characteristics of patients with severe asynchrony

Nine patients were considered as severely asynchronous, with a proportion of time spent in asynchrony > 75th percentile, i.e., > 39% of time (Table 2). Patients with severe asynchrony were younger (p = 0.007), had more frequently a narrower and noncuffed ETT (p = 0.001 and p = 0.019, respectively), and were less frequently ventilated in pressure-support ventilation (PSV, p = 0.034). All but one of these patients were admitted for a respiratory failure as a first reason, and five of them had bronchiolitis. In the multivariate logistic regression model in which age, presence of a cuffed ETT, and PSV mode were tested, none of these variables were independently associated with severe PVA (all p > 0.17).

**Table 2:** Characteristics of patients depending on the level of asynchrony (in patients with Edi > 2 µV, *n* = 34)

	% time spent in asynchrony < 39% (*n* = 25)	% time spent in asynchrony > 39% (*n* = 9)	p value
Age (m)	14 (2–40)	2 (1–3)	0.007
Weight (kg)	7.0 (4.5–17.3)	4.3 (3.6–5.4)	0.049
Male, *n* (%)	14 (56%)	6 (67%)	0.70
Days between admission and inclusion	8 (2–11)	2 (1–5)	0.054
Main reasons for PICU admission, *n* (%)			0.56
Respiratory failure	18 (72%)	8 (89%)	0.40
Including bronchiolitis	5 (20%)	5 (56%)	0.08
Hemodynamic failure	1 (4%)	0 (0%)	1
Neurologic disorder	3 (12%)	0 (0%)	0.55
Metabolic disorder	1 (4%)	1 (11%)	0.46
Trauma	0 (0%)	0 (0%)	1
Post-surgery	2 (8%)	0 (0%)	1
Chronic condition, *n* (%)			
Respiratory disease	5 (20%)	1 (11%)	1
Cardiac disease	6 (24%)	0 (0%)	0.16
Neurological disease	6 (24%)	1 (11%)	0.64
Immuno-oncologic disease	3 (12%)	0 (0%)	0.55
Clinical status			
PIM-2 score	2.5 (0.9–4.4)	0.9 (0.5–7.0)	0.40
PELOD score	1 (1–11)	11 (1–12)	0.38
pH	7.40 (7.33–7.42)	7.37 (7.33–7.42)	0.63
HCO_3_^−^, mmHg	30.0 (25.1–32.9)	28.8 (24.9–32.0)	0.84
PaCO_2_, mmHg	48.0 (44.4–53.4)	48.9 (45.8–57.5)	0.57
Hb, g/dL	10.2 (7.3–10.7)	10.4 (7.9–12.3)	0.33
Lactate, mmol/L	1.5 (0.8–2.1)	1.5 (1.2–1.9)	1
Comfort score	15 (13–16)	15 (11–17)	0.95
Sedation score	11 (6–23)	21 (11–39)	0.15
ETT size	4.0 (3.5–4.5)	3.5 (3.5–3.5)	0.013
Cuffed ETT	17 (68%)	2 (22%)	0.019
Ventilatory settings			
Set RR	25 (20–35)	30 (28–38)	0.13
Measured RR	34 (28–40)	35 (29–40)	0.92
Mode PSV	10 (40%)	0 (0%)	0.034
Mode ACV-P	4 (16%)	3 (33%)	0.35
Mode IACV-P	7 (28%)	3 (33%)	1
Mode ACV-V	0 (0%)	0 (0%)	1
Mode IACV-V	1 (4%)	2 (22%)	0.16
Mode PRVC	3 (12%)	1 (11%)	1
PEEP, cmH_2_O	5 (5–5)	6 (5–7)	0.06
FiO_2_	0.35 (0.26–0.44)	0.35 (0.30–0.60)	0.45
Leaks (%)	7 (4–15)	2 (0–7)	0.17
Analysis			
Peak inspiratory Edi, µV	7.2 (3.8–15.3)	5.5 (3.4–7.2)	0.20
Tonic expiratory Edi, µV	0.9 (0.6–2.4)	2.0 (1.1–2.9)	0.058
Type of asynchrony			
Wasted Efforts, % of breath analyzed	4.5 (1.6–15.8)	30.6 (18.7–39.8)	0.002
Auto-triggering, % of breath analyzed	6.1 (1.3–9.9)	8.4 (0.9–23.3)	0.36
Double triggering, % of breath analyzed	2.1 (0.0–3.2)	0.0 (0.0–0.8)	0.08
Trigger error, ms	136 (104–176)	284 (190–302)	0.008
Cycling-off error, ms	64 (40–131)	255 (184–297)	0.018
Time spent in asynchrony			
Total time spent in asynchrony, %	24 (17–28)	47 (43–50)	< 0.001
Wasted Effort, %	0.6 (0.2–3.5)	5.3 (2.8–13.6)	0.03
Auto-triggering, %	1.6 (0.3–2.4)	2.3 (0.3–4.7)	0.40
Double triggering, %	0.1 (0.0–0.4)	0.0 (0.0–0.1)	0.053
*Trigger error*			
Delay, %	7.6 (7.6–11.2)	15.5 (12.2–19.1)	0.001
Premature, %	0.8 (0.5–2.1)	2.3 (1.4–2.9)	0.058
*Cycle*-*off error*			
Delay, %	3.8 (1.8–6.3)	15.0 (10.2–17.5)	< 0.001
Premature, %	4.1 (2.2–5.9)	3.2 (2.0–6.7)	0.98
NeuroSync index, %	38 (31–47)	81 (69–83)	< 0.001
Outcome			
Death in PICU	1 (4.0%)	1 (11.1%)	1
Days in PICU	14 (5–22)	7 (4–14)	0.17
Days in PICU after inclusion	6 (4–12.5)	5 (3–6)	0.66
Days on MV	9 (4–15)	4 (3–12)	0.23
Days on MV after inclusion	2.5 (1–6.5)	3 (1–4)	0.9
NIV post extubation	4 (16.0%)	1 (11.1%)	1
Reintubation	5 (20.0%)	1 (11.1%)	1

*Edi* electrical activity of the diaphragm, *PICU* pediatric intensive care unit, *RR* respiratory rate, *PSV* pressure-support ventilation, *ACV-P* pressure-regulated assist control ventilation, *IACV-P* pressure-regulated intermittent assist control ventilation, *ACV-V* volume-regulated assist control ventilation, *IACV-V* volume-regulated intermittent assist control ventilation, *PRVC* pressure-regulated volume control ventilation, *PEEP* positive end-expiratory pressure, *ETT* endo-tracheal tube, *MV* mechanical ventilation, *NIV* noninvasive ventilation

The patients with severe asynchrony were enrolled earlier in the PICU course (2 days (1–5) vs 8 (2–11), p = 0.054), which must be considered while looking at the relationship between PVA and length of stay or ventilation duration.

### Evolution of PVA

**Fig. 3:**
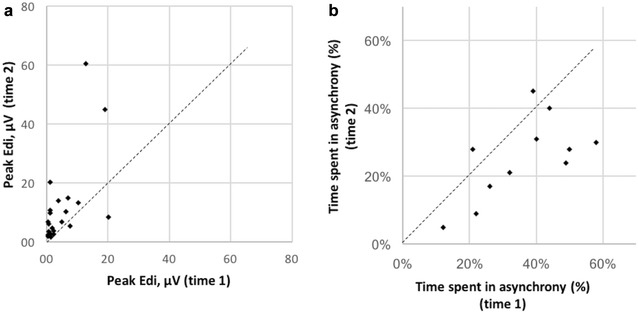
Evolution of inspiratory Edi (panel a) and of the time spent in asynchrony (panel b) from inclusion time (time 1) to pre-extubation period (time 2)

As illustrated in Fig. 3, when comparing the recordings from acute phase and pre-extubation phase, the level of PVA tended to decrease over time (p = 0.01), and both period data were correlated (*R* ^2^ = 0.41). Peak Edi increased between the two phases (p = 0.01).

### Automatic analysis of PVA

**Fig. 4:**
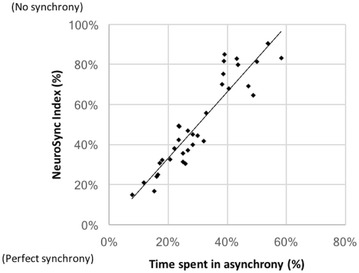
Relationship between the asynchrony results obtained using the two methods: the automatic NeuroSync index and the percentage of time spent in asynchrony derived from the manual breath-by-breath analysis

The automatic algorithm provided a NeuroSync index of 45% (32–70%), confirming the high prevalence of asynchrony. As shown in Fig. 4, a good correlation was observed between NeuroSync index and the percentage of time spent in asynchrony derived from the manual analysis, with an ICC of 0.88.

### Outcome

We did not observe any association between the level of asynchrony and neither ventilator-free days at day 28, nor the secondary outcomes (Table 2). This holds true with the manual classification as severe PVA or not (Table 2), as well as with the automated NeuroSync index (correlation with ventilation duration: *R* ^2^ = 0.12; p = 0.58). None of the patient characteristics were associated with the duration of mechanical ventilation.

## Discussion

The incidence of PVA is very high during pediatric conventional ventilation. As a whole, children spend about one-third of the time in conflict with their ventilator. We described an a priori defined group with severe PVA, but marked PVA was present even in the other children, and the proportion of children which could be considered as “well synchronized” is low. Besides, an unexpected form of bad interaction was observed, with the high prevalence of low ventilatory drive.

The magnitude of PVA that we observed is in agreement with that previously described [10–12]. In a recent study conducted in a PICU, Blokpoel et al. [10] showed that PVA occurred in 33% of breaths. These authors identified PVA using the analysis of ventilator waveforms, a method which has a low sensitivity [6]. We used the Edi signal which clearly facilitates the detection of PVA, in particular the calculation of timing errors for triggering or cycling off [3, 12, 13, 17, 22, 23]. We were therefore able to show that most of the time spent in asynchrony results from delayed or premature reactions of the ventilator. These timing errors are important, especially when the normal inspiratory time is frequently around 400 ms in this population. We hypothesize that this delay in ventilator response is the consequence of small tidal volumes and short inspiratory and expiratory times in children as compared to adults. Although considered as the classical method [12, 17, 24], the breath-by-breath manual analysis of PVA could be criticized because of its dependency on an investigator, as well as being highly time consuming. However, our findings were supported by the good agreement between the two independent investigators, and by the concordance also observed between the automatically calculated NeuroSync index and the manually calculated PVA.

To date, no definition of severe PVA in children had been standardized. Some authors use the specific index described in adults by Thille et al. [5, 25] and others the percentage of asynchronous breaths [3, 10, 12]. In the present study, we primarily assessed the magnitude of PVA according to the time spent in asynchrony, because it illustrates well the burden of asynchrony while taking into account different types of patient–ventilator conflict [17]. As expressed using the AI, our results confirm the severity of PVA, a huge proportion of the patients having an AI > 10%. A recent meta-analysis reported that the mean reported AI varied from 13 to 37% in adults, and from 38 to 74% in children during conventional ventilation, while a significant decrease in AI was observed with NAVA [18]. Consistent with the other PVA indices, only two patients in our series had a NeuroSync index < 20%, which corresponds to an adequate synchrony [20, 21]. The nonsevere group can therefore not be assumed as “well synchronized.” In agreement with Blokpoel et al. [10], who observed that only 20% children had an acceptable level of PVA, our study highlights that PVA is a major problem in PICU and concerns more than three-quarters of the children, as opposed to one-quarter of adult patients.

Younger age, smaller tracheal tubes, and absence of a cuff on the tracheal tube were associated with severe PVA, and PSV mode was more frequent in patients with less severe PVA. The smaller size and the absence of cuff may suggest that increased leaks could have played a role, as suggested by Blokpoel et al. [10]. The magnitude of the leaks was not different between the two groups, but the precision of this measure is not perfect [26]. None of the patients ventilated in PSV was classified as severe PVA. We may hypothesize that the patients ventilated in PSV have a stronger ventilatory drive, leading to a better detection of the breathing efforts by the ventilator [5]. However, a confounding factor may also explain this association, PSV being mostly used in our unit in older and less sedated patients.

Overall, we did not observe any association between severe asynchrony and adverse outcomes during the PICU course, in contrast to studies in adults [4, 5, 7]. Similarly, Blokpoel et al. did not observe prolonged ventilation in patients with higher levels of asynchrony. While this may be the consequence of the limited power of these two pediatric studies, several explanations could be hypothesized to explain this difference with adult studies. In adults, adverse outcome was observed in severe PVA groups, while the remaining patients were appropriately synchronized [4, 5, 7]. In contrast, the number of children with good patient–ventilator interaction is quite low. In our study, patients with severe PVA frequently had diseases usually associated with good outcome (e.g., bronchiolitis). It is also important to note that the patients with more severe PVA were recorded earlier in the PICU course. This baseline discrepancy makes it difficult to assess the relationship between PVA and ventilation duration.

The question remains whether those children would have a better outcome providing the PVA was improved. Only a controlled interventional trial, for example using a specific mode like NAVA, could confirm the independent role of PVA on outcome. Such evidence remains limited in PICU. In a crossover trial conducted in 12 children, De la Oliva et al. [13] observed that the improvement in PVA with NAVA was associated with an improvement in comfort score. This has been supported by another study by Piastra et al. [27]. This finding is interesting when sedation is sometimes needed in cases of severe asynchrony. In the present study, we were not able to confirm that a better synchrony leads to a better comfort for the patient, as similar comfort score was observed in both groups. However, the patient with sever asynchrony tended to require more sedatives, as illustrated by higher sedation score (21 (11–39) vs 11 (6–23), although this difference did not reach significance (p = 0.15). An improved synchrony might have the potential to reduce sedation needs and its associated side effects. In a large randomized controlled trial, Kallio et al. [28] observed an interesting trend for shorter ventilation and ICU length of stay using NAVA (p = 0.03 and p = 0.07, respectively).

Finally, some authors hypothesize that improved PVA could also have deleterious effects that counterbalance the benefits [29]. It is, however, difficult to retain this hypothesis here while very few patients had good synchrony.

Interestingly, the peak Edi in the present study was relatively low (IQR 1.2–7.6) as compared to values observed in extubated children, which usually are between 5 and 30 mcV, depending on the lung condition [30, 31]. Many patients had low respiratory drive after several days of intubation, while they were deemed to be actively breathing. We consider this finding as a new form of poor patient–ventilator interaction, although not an asynchrony. This low respiratory activity has previously been reported [14, 32]. It could be the consequence of overassistance, oversedation, their combination, or more rarely of an abnormal output by the central respiratory center or by bilateral phrenic nerve palsy [33, 34]. In this study group, many patients were admitted for nonrespiratory reasons. Even low level of ventilator support can be sufficient in such conditions to suppress the patient breaths [35]. We previously reported that the ventilatory drive increased in these patients after the extubation, so the central or peripheral neurological explanation seems unlikely [31]. Oversedation may have contributed, as suggested by higher degree of comfort observed in these patients. As described by Vaschetto et al., the combination of overassistance and sedation has a synergistic impact on the drive suppression. More attention should be paid to this frequent complication, especially since such respiratory behavior has clearly been linked to diaphragm dysfunction [30, 36, 37].

Several limitations of our study need to be discussed. We included in the analysis fewer patients than expected. It is possible that our study was underpowered in particular to conduct subgroup analysis or to truly assess the impact on patient outcome. This is a single-center study, and the results may have been influenced by the local practice, especially regarding ventilator settings. NAVA was not used in routine practice during the study period in our PICU. NAVA can improve patient–ventilator interactions [12, 18, 38], and the results of our study would probably be different in population treated with this mode. Many patients were not included, which could limit the external validity of our findings. Certain medical conditions, as chronic respiratory insufficiency with prior ventilatory support, tracheostomy or neuromuscular disease, were a priori excluded, preventing us to generalize our findings to these patients. Two ventilators (Evita XL and Servo-I) were used during the study. Similar studies are necessary to confirm our findings with other ventilators. Due to the study design and the need to observe active breathing for considering patient inclusion, patients were not recorded at the same time after admission. Although the degree of PVA did not seem to change so much over the PICU course, this difference in inclusion timing made it difficult to interpret the relationship between asynchrony and outcome.

## Conclusion

Patient–ventilator interaction is poor in critically ill children supported by conventional ventilation. The study did not permit to ascertain if these poor interactions have important clinical consequence. But the magnitude of PVA and the prevalence of low ventilatory drive warrant further studies to assess whether strategies to optimize patient–ventilator interactions can improve the outcome of PICU patients.

## Abbreviations

Edi: electrical activity of the diaphragm
ETT: endotracheal tube
NAVA: neurally adjusted ventilatory assist
PICU: pediatric intensive care unit
PSV: pressure support ventilation
PVA: patient–ventilator asynchrony
